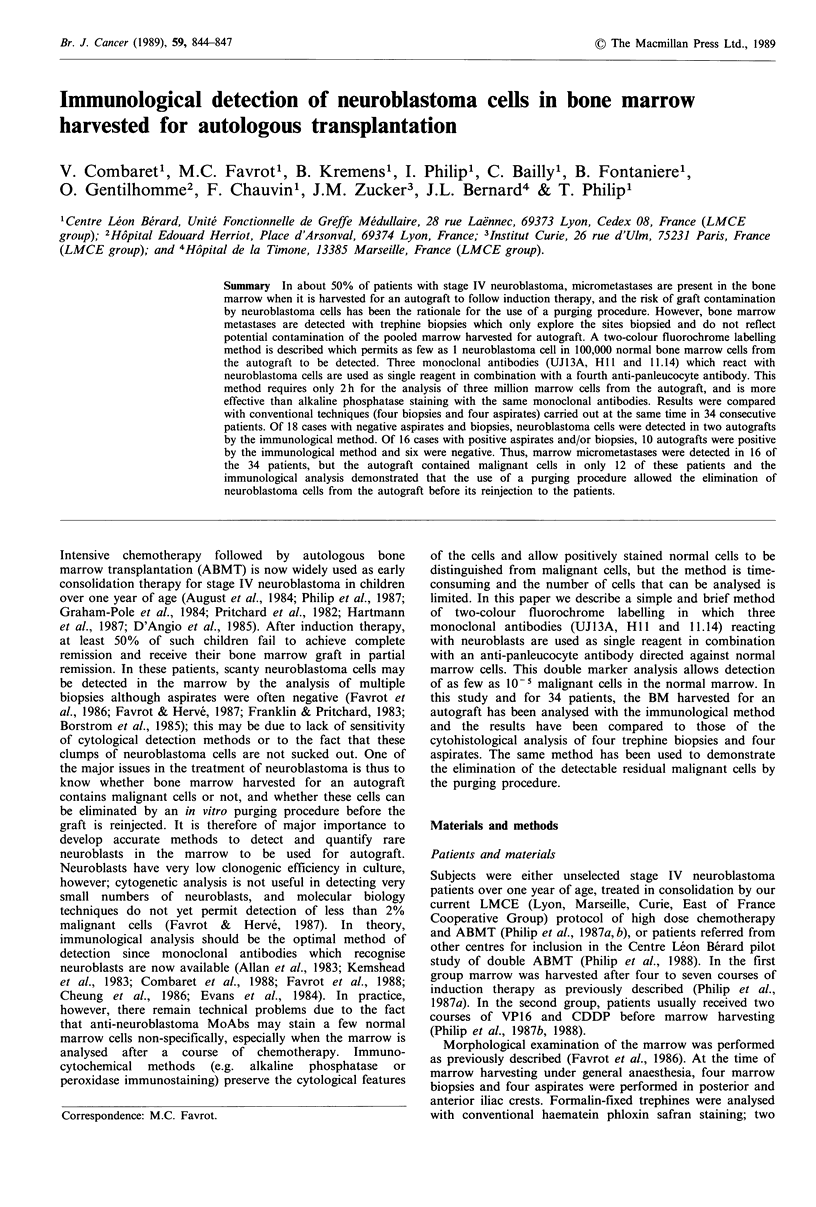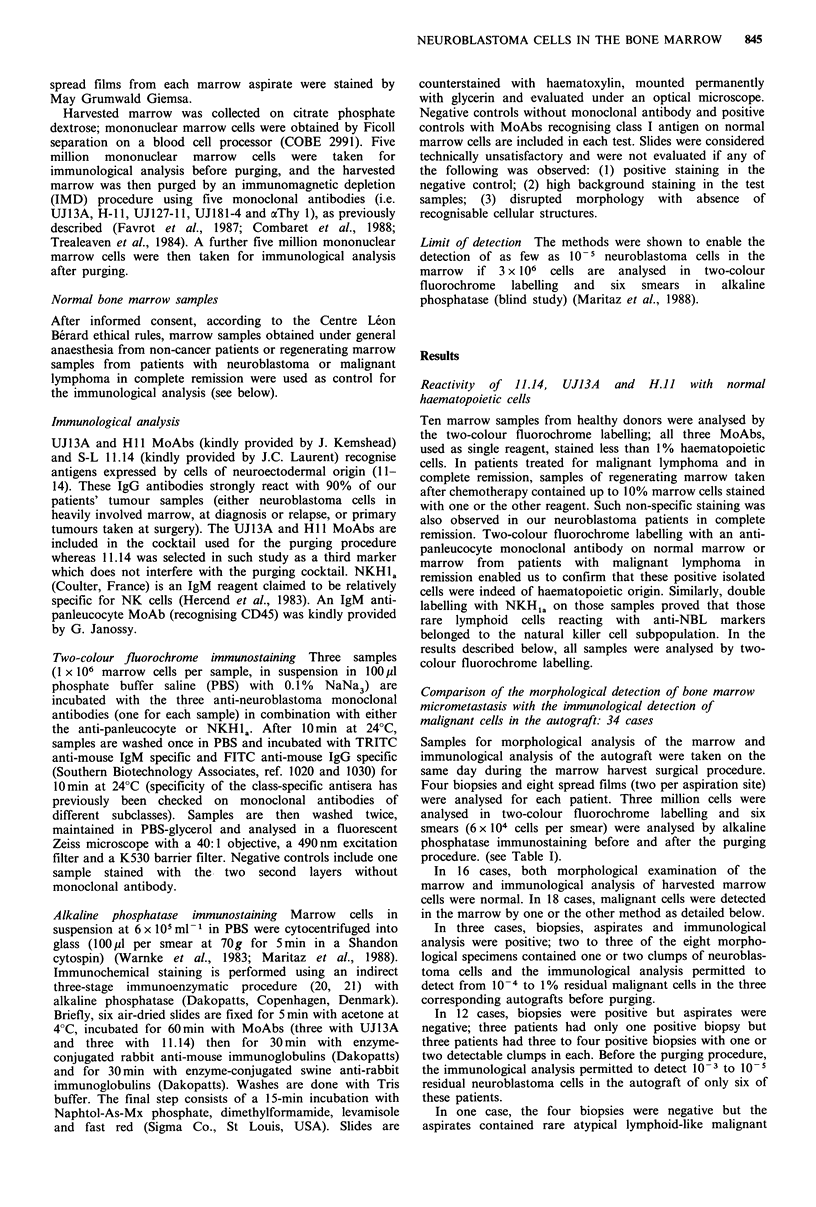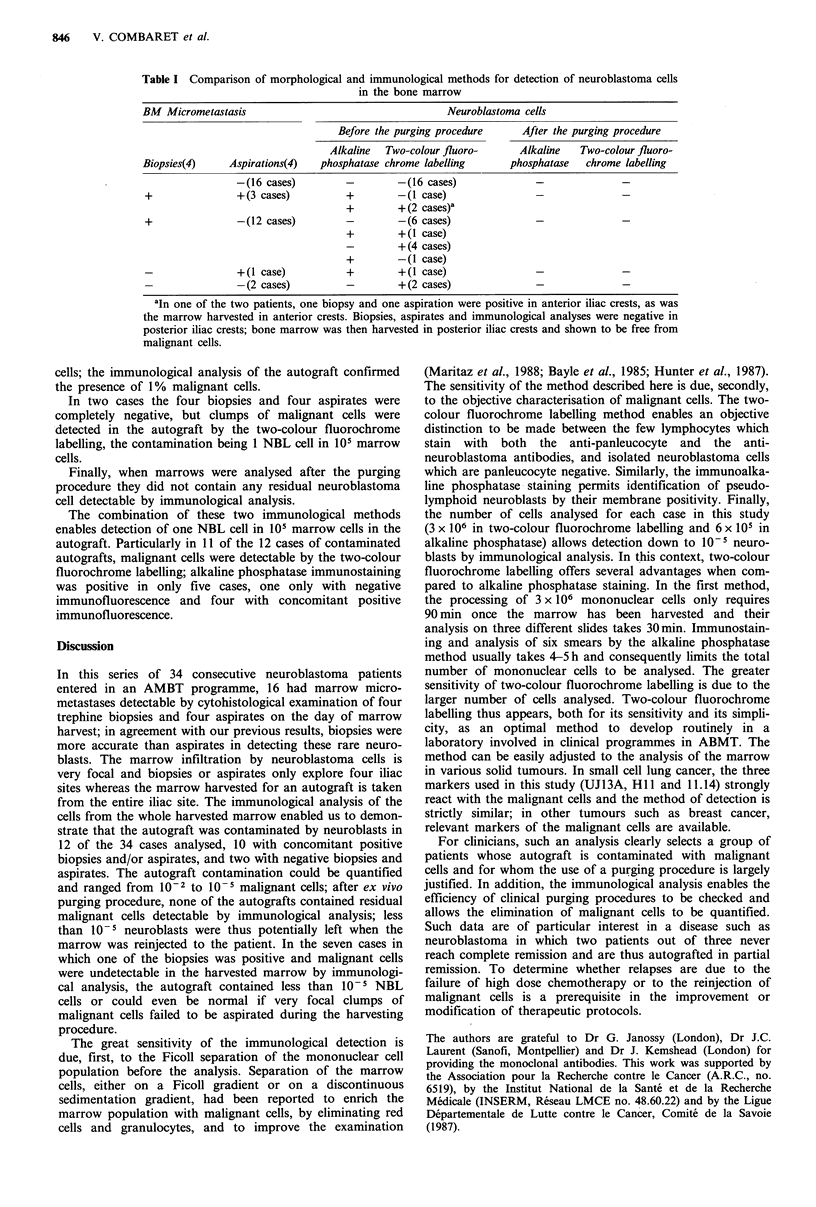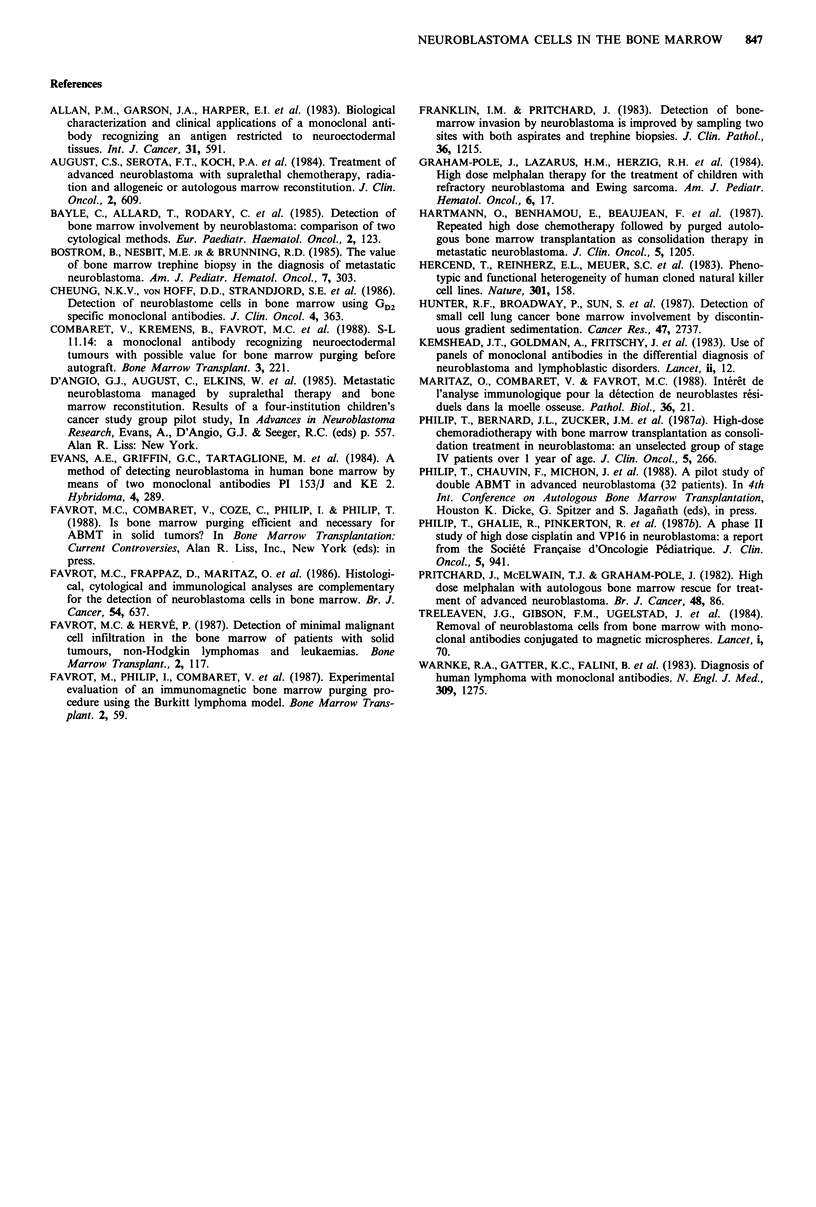# Immunological detection of neuroblastoma cells in bone marrow harvested for autologous transplantation.

**DOI:** 10.1038/bjc.1989.180

**Published:** 1989-06

**Authors:** V. Combaret, M. C. Favrot, B. Kremens, I. Philip, C. Bailly, B. Fontaniere, O. Gentilhomme, F. Chauvin, J. M. Zucker, J. L. Bernard

**Affiliations:** Centre LÃ©on BÃ©rard, UnitÃ© Fonctionnelle de Greffe MÃ©dullaire, Lyon, France.

## Abstract

In about 50% of patients with stage IV neuroblastoma, micrometastases are present in the bone marrow when it is harvested for an autograft to follow induction therapy, and the risk of graft contamination by neuroblastoma cells has been the rationale for the use of a purging procedure. However, bone marrow metastases are detected with trephine biopsies which only explore the sites biopsied and do not reflect potential contamination of the pooled marrow harvested for autograft. A two-colour fluorochrome labelling method is described which permits as few as 1 neuroblastoma cell in 100,000 normal bone marrow cells from the autograft to be detected. Three monoclonal antibodies (UJ13A, H11 and 11.14) which react with neuroblastoma cells are used as single reagent in combination with a fourth anti-panleucocyte antibody. This method requires only 2 h for the analysis of three million marrow cells from the autograft, and is more effective than alkaline phosphatase staining with the same monoclonal antibodies. Results were compared with conventional techniques (four biopsies and four aspirates) carried out at the same time in 34 consecutive patients. Of 18 cases with negative aspirates and biopsies, neuroblastoma cells were detected in two autografts by the immunological method. Of 16 cases with positive aspirates and/or biopsies, 10 autografts were positive by the immunological method and six were negative. Thus, marrow micrometastases were detected in 16 of the 34 patients, but the autograft contained malignant cells in only 12 of these patients and the immunological analysis demonstrated that the use of a purging procedure allowed the elimination of neuroblastoma cells from the autograft before its reinjection to the patients.


Br. J. Cancer (1989), 59, 844–847 © The Macmillan Press Ltd., 1989

### Immunological detection of neuroblastoma cells in bone marrow harvested for autologous transplantation

V. Combaret[1], M.C. Favrot[1], B. Kremens[1], I. Philip[1], C. Bailly[1], B. Fontaniere[1], O. Gentilhomme[2], F. Chauvin[1], J.M. Zucker[3], J.L. Bernard[4] & T. Philip[1]

[1]*Centre Léon Bérard, Unité Fonctionnelle de Greffe Médullaire, 28 rue Laënnec, 69373 Lyon, Cedex 08, France (LMCE group);* [2]*Hôpital Edouard Herriot, Place d'Arsonval, 69374 Lyon, France;* [3]*Institut Curie, 26 rue d'Ulm, 75231 Paris, France (LMCE group); and* [4]*Hôpital de la Timone, 13385 Marseille, France (LMCE group).*

**Summary** In about 50% of patients with stage IV neuroblastoma, micrometastases are present in the bone marrow when it is harvested for an autograft to follow induction therapy, and the risk of graft contamination by neuroblastoma cells has been the rationale for the use of a purging procedure. However, bone marrow metastases are detected with trephine biopsies which only explore the sites biopsied and do not reflect potential contamination of the pooled marrow harvested for autograft. A two-colour fluorochrome labelling method is described which permits as few as 1 neuroblastoma cell in 100,000 normal bone marrow cells from the autograft to be detected. Three monoclonal antibodies (UJ13A, H11 and 11.14) which react with neuroblastoma cells are used as single reagent in combination with a fourth anti-panleucocyte antibody. This method requires only 2 h for the analysis of three million marrow cells from the autograft, and is more effective than alkaline phosphatase staining with the same monoclonal antibodies. Results were compared with conventional techniques (four biopsies and four aspirates) carried out at the same time in 34 consecutive patients. Of 18 cases with negative aspirates and biopsies, neuroblastoma cells were detected in two autografts by the immunological method. Of 16 cases with positive aspirates and/or biopsies, 10 autografts were positive by the immunological method and six were negative. Thus, marrow micrometastases were detected in 16 of the 34 patients, but the autograft contained malignant cells in only 12 of these patients and the immunological analysis demonstrated that the use of a purging procedure allowed the elimination of neuroblastoma cells from the autograft before its reinjection to the patients.

Intensive chemotherapy followed by autologous bone marrow transplantation (ABMT) is now widely used as early consolidation therapy for stage IV neuroblastoma in children over one year of age (August *et al.*, 1984; Philip *et al.*, 1987; Graham-Pole *et al.*, 1984; Pritchard *et al.*, 1982; Hartmann *et al.*, 1987; D'Angio *et al.*, 1985). After induction therapy, at least 50% of such children fail to achieve complete remission and receive their bone marrow graft in partial remission. In these patients, scanty neuroblastoma cells may be detected in the marrow by the analysis of multiple biopsies although aspirates were often negative (Favrot *et al.*, 1986; Favrot & Hervé, 1987; Franklin & Pritchard, 1983; Borstrom *et al.*, 1985); this may be due to lack of sensitivity of cytological detection methods or to the fact that these clumps of neuroblastoma cells are not sucked out. One of the major issues in the treatment of neuroblastoma is thus to know whether bone marrow harvested for an autograft contains malignant cells or not, and whether these cells can be eliminated by an *in vitro* purging procedure before the graft is reinjected. It is therefore of major importance to develop accurate methods to detect and quantify rare neuroblasts in the marrow to be used for autograft. Neuroblasts have very low clonogenic efficiency in culture, however; cytogenetic analysis is not useful in detecting very small numbers of neuroblasts, and molecular biology techniques do not yet permit detection of less than 2% malignant cells (Favrot & Hervé, 1987). In theory, immunological analysis should be the optimal method of detection since monoclonal antibodies which recognise neuroblasts are now available (Allan *et al.*, 1983; Kemshead *et al.*, 1983; Combaret *et al.*, 1988; Favrot *et al.*, 1988; Cheung *et al.*, 1986; Evans *et al.*, 1984). In practice, however, there remain technical problems due to the fact that anti-neuroblastoma MoAbs may stain a few normal marrow cells non-specifically, especially when the marrow is analysed after a course of chemotherapy. Immuno-cytochemical methods (e.g. alkaline phosphatase or peroxidase immunostaining) preserve the cytological features of the cells and allow positively stained normal cells to be distinguished from malignant cells, but the method is time-consuming and the number of cells that can be analysed is limited. In this paper we describe a simple and brief method of two-colour fluorochrome labelling in which three monoclonal antibodies (UJ13A, H11 and 11.14) reacting with neuroblasts are used as single reagent in combination with an anti-panleucocyte antibody directed against normal marrow cells. This double marker analysis allows detection of as few as $10^{-5}$ malignant cells in the normal marrow. In this study and for 34 patients, the BM harvested for an autograft has been analysed with the immunological method and the results have been compared to those of the cytohistological analysis of four trephine biopsies and four aspirates. The same method has been used to demonstrate the elimination of the detectable residual malignant cells by the purging procedure.

Correspondence: M.C. Favrot.

### Materials and methods

### *Patients and materials*

Subjects were either unselected stage IV neuroblastoma patients over one year of age, treated in consolidation by our current LMCE (Lyon, Marseille, Curie, East of France Cooperative Group) protocol of high dose chemotherapy and ABMT (Philip *et al.*, 1987*a*, *b*), or patients referred from other centres for inclusion in the Centre Léon Bérard pilot study of double ABMT (Philip *et al.*, 1988). In the first group marrow was harvested after four to seven courses of induction therapy as previously described (Philip *et al.*, 1987*a*). In the second group, patients usually received two courses of VP16 and CDDP before marrow harvesting (Philip *et al.*, 1987*b*, 1988).

Morphological examination of the marrow was performed as previously described (Favrot *et al.*, 1986). At the time of marrow harvesting under general anaesthesia, four marrow biopsies and four aspirates were performed in posterior and anterior iliac crests. Formalin-fixed trephines were analysed with conventional haematein phloxin safran staining; two spread films from each marrow aspirate were stained by May Grumwald Giemsa.

Harvested marrow was collected on citrate phosphate dextrose; mononuclear marrow cells were obtained by Ficoll separation on a blood cell processor (COBE 2991). Five million mononuclear marrow cells were taken for immunological analysis before purging, and the harvested marrow was then purged by an immunomagnetic depletion (IMD) procedure using five monoclonal antibodies (i.e. UJ13A, H-11, UJ127-11, UJ181-4 and αThy 1), as previously described (Favrot *et al.*, 1987; Combaret *et al.*, 1988; Trealeaven *et al.*, 1984). A further five million mononuclear marrow cells were then taken for immunological analysis after purging.

*Normal bone marrow samples*

After informed consent, according to the Centre Léon Bérard ethical rules, marrow samples obtained under general anaesthesia from non-cancer patients or regenerating marrow samples from patients with neuroblastoma or malignant lymphoma in complete remission were used as control for the immunological analysis (see below).

*Immunological analysis*

UJ13A and H11 MoAbs (kindly provided by J. Kemshead) and S-L 11.14 (kindly provided by J.C. Laurent) recognise antigens expressed by cells of neuroectodermal origin (11–14). These IgG antibodies strongly react with 90% of our patients' tumour samples (either neuroblastoma cells in heavily involved marrow, at diagnosis or relapse, or primary tumours taken at surgery). The UJ13A and H11 MoAbs are included in the cocktail used for the purging procedure whereas 11.14 was selected in such study as a third marker which does not interfere with the purging cocktail. $NKH1_a$ (Coulter, France) is an IgM reagent claimed to be relatively specific for NK cells (Hercend *et al.*, 1983). An IgM anti-panleucocyte MoAb (recognising CD45) was kindly provided by G. Janossy.

*Two-colour fluorochrome immunostaining* Three samples ($1 \times 10^6$ marrow cells per sample, in suspension in 100 µl phosphate buffer saline (PBS) with 0.1% $NaNa_3$) are incubated with the three anti-neuroblastoma monoclonal antibodies (one for each sample) in combination with either the anti-panleucocyte or $NKH1_a$. After 10 min at 24°C, samples are washed once in PBS and incubated with TRITC anti-mouse IgM specific and FITC anti-mouse IgG specific (Southern Biotechnology Associates, ref. 1020 and 1030) for 10 min at 24°C (specificity of the class-specific antisera has previously been checked on monoclonal antibodies of different subclasses). Samples are then washed twice, maintained in PBS-glycerol and analysed in a fluorescent Zeiss microscope with a 40:1 objective, a 490 nm excitation filter and a K530 barrier filter. Negative controls include one sample stained with the two second layers without monoclonal antibody.

*Alkaline phosphatase immunostaining* Marrow cells in suspension at $6 \times 10^5\,ml^{-1}$ in PBS were cytocentrifuged into glass (100 µl per smear at 70*g* for 5 min in a Shandon cytospin) (Warnke *et al.*, 1983; Maritaz *et al.*, 1988). Immunochemical staining is performed using an indirect three-stage immunoenzymatic procedure (20, 21) with alkaline phosphatase (Dakopatts, Copenhagen, Denmark). Briefly, six air-dried slides are fixed for 5 min with acetone at 4°C, incubated for 60 min with MoAbs (three with UJ13A and three with 11.14) then for 30 min with enzyme-conjugated rabbit anti-mouse immunoglobulins (Dakopatts) and for 30 min with enzyme-conjugated swine anti-rabbit immunoglobulins (Dakopatts). Washes are done with Tris buffer. The final step consists of a 15-min incubation with Naphtol-As-Mx phosphate, dimethylformamide, levamisole and fast red (Sigma Co., St Louis, USA). Slides are counterstained with haematoxylin, mounted permanently with glycerin and evaluated under an optical microscope. Negative controls without monoclonal antibody and positive controls with MoAbs recognising class I antigen on normal marrow cells are included in each test. Slides were considered technically unsatisfactory and were not evaluated if any of the following was observed: (1) positive staining in the negative control; (2) high background staining in the test samples; (3) disrupted morphology with absence of recognisable cellular structures.

*Limit of detection* The methods were shown to enable the detection of as few as $10^{-5}$ neuroblastoma cells in the marrow if $3 \times 10^6$ cells are analysed in two-colour fluorochrome labelling and six smears in alkaline phosphatase (blind study) (Maritaz *et al.*, 1988).

### Results

*Reactivity of 11.14, UJ13A and H.11 with normal haematopoietic cells*

Ten marrow samples from healthy donors were analysed by the two-colour fluorochrome labelling; all three MoAbs, used as single reagent, stained less than 1% haematopoietic cells. In patients treated for malignant lymphoma and in complete remission, samples of regenerating marrow taken after chemotherapy contained up to 10% marrow cells stained with one or the other reagent. Such non-specific staining was also observed in our neuroblastoma patients in complete remission. Two-colour fluorochrome labelling with an anti-panleucocyte monoclonal antibody on normal marrow or marrow from patients with malignant lymphoma in remission enabled us to confirm that these positive isolated cells were indeed of haematopoietic origin. Similarly, double labelling with $NKH_{1a}$ on those samples proved that those rare lymphoid cells reacting with anti-NBL markers belonged to the natural killer cell subpopulation. In the results described below, all samples were analysed by two-colour fluorochrome labelling.

*Comparison of the morphological detection of bone marrow micrometastasis with the immunological detection of malignant cells in the autograft: 34 cases*

Samples for morphological analysis of the marrow and immunological analysis of the autograft were taken on the same day during the marrow harvest surgical procedure. Four biopsies and eight spread films (two per aspiration site) were analysed for each patient. Three million cells were analysed in two-colour fluorochrome labelling and six smears ($6 \times 10^4$ cells per smear) were analysed by alkaline phosphatase immunostaining before and after the purging procedure. (see Table I).

In 16 cases, both morphological examination of the marrow and immunological analysis of harvested marrow cells were normal. In 18 cases, malignant cells were detected in the marrow by one or the other method as detailed below.

In three cases, biopsies, aspirates and immunological analysis were positive; two to three of the eight morphological specimens contained one or two clumps of neuroblastoma cells and the immunological analysis permitted to detect from $10^{-4}$ to 1% residual malignant cells in the three corresponding autografts before purging.

In 12 cases, biopsies were positive but aspirates were negative; three patients had only one positive biopsy but three patients had three to four positive biopsies with one or two detectable clumps in each. Before the purging procedure, the immunological analysis permitted to detect $10^{-3}$ to $10^{-5}$ residual neuroblastoma cells in the autograft of only six of these patients.

In one case, the four biopsies were negative but the aspirates contained rare atypical lymphoid-like malignant

**Table I** Comparison of morphological and immunological methods for detection of neuroblastoma cells in the bone marrow

| *BM Micrometastasis* | | *Neuroblastoma cells* | | | |
|---|---|---|---|---|---|
| | | *Before the purging procedure* | | *After the purging procedure* | |
| *Biopsies(4)* | *Aspirations(4)* | *Alkaline phosphatase* | *Two-colour fluorochrome labelling* | *Alkaline phosphatase* | *Two-colour fluorochrome labelling* |
| | −(16 cases) | − | −(16 cases) | − | − |
| + | +(3 cases) | + | −(1 case) | − | − |
| | | + | +(2 cases)[a] | | |
| + | −(12 cases) | − | −(6 cases) | − | − |
| | | + | +(1 case) | | |
| | | − | +(4 cases) | | |
| | | + | −(1 case) | | |
| − | +(1 case) | + | +(1 case) | − | − |
| − | −(2 cases) | − | +(2 cases) | − | − |

[a]In one of the two patients, one biopsy and one aspiration were positive in anterior iliac crests, as was the marrow harvested in anterior crests. Biopsies, aspirates and immunological analyses were negative in posterior iliac crests; bone marrow was then harvested in posterior iliac crests and shown to be free from malignant cells.

cells; the immunological analysis of the autograft confirmed the presence of 1% malignant cells.

In two cases the four biopsies and four aspirates were completely negative, but clumps of malignant cells were detected in the autograft by the two-colour fluorochrome labelling, the contamination being 1 NBL cell in $10^5$ marrow cells.

Finally, when marrows were analysed after the purging procedure they did not contain any residual neuroblastoma cell detectable by immunological analysis.

The combination of these two immunological methods enables detection of one NBL cell in $10^5$ marrow cells in the autograft. Particularly in 11 of the 12 cases of contaminated autografts, malignant cells were detectable by the two-colour fluorochrome labelling; alkaline phosphatase immunostaining was positive in only five cases, one only with negative immunofluorescence and four with concomitant positive immunofluorescence.

### Discussion

In this series of 34 consecutive neuroblastoma patients entered in an AMBT programme, 16 had marrow micrometastases detectable by cytohistological examination of four trephine biopsies and four aspirates on the day of marrow harvest; in agreement with our previous results, biopsies were more accurate than aspirates in detecting these rare neuroblasts. The marrow infiltration by neuroblastoma cells is very focal and biopsies or aspirates only explore four iliac sites whereas the marrow harvested for an autograft is taken from the entire iliac site. The immunological analysis of the cells from the whole harvested marrow enabled us to demonstrate that the autograft was contaminated by neuroblasts in 12 of the 34 cases analysed, 10 with concomitant positive biopsies and/or aspirates, and two with negative biopsies and aspirates. The autograft contamination could be quantified and ranged from $10^{-2}$ to $10^{-5}$ malignant cells; after *ex vivo* purging procedure, none of the autografts contained residual malignant cells detectable by immunological analysis; less than $10^{-5}$ neuroblasts were thus potentially left when the marrow was reinjected to the patient. In the seven cases in which one of the biopsies was positive and malignant cells were undetectable in the harvested marrow by immunological analysis, the autograft contained less than $10^{-5}$ NBL cells or could even be normal if very focal clumps of malignant cells failed to be aspirated during the harvesting procedure.

The great sensitivity of the immunological detection is due, first, to the Ficoll separation of the mononuclear cell population before the analysis. Separation of the marrow cells, either on a Ficoll gradient or on a discontinuous sedimentation gradient, had been reported to enrich the marrow population with malignant cells, by eliminating red cells and granulocytes, and to improve the examination (Maritaz *et al.*, 1988; Bayle *et al.*, 1985; Hunter *et al.*, 1987). The sensitivity of the method described here is due, secondly, to the objective characterisation of malignant cells. The two-colour fluorochrome labelling method enables an objective distinction to be made between the few lymphocytes which stain with both the anti-panleucocyte and the anti-neuroblastoma antibodies, and isolated neuroblastoma cells which are panleucocyte negative. Similarly, the immunoalkaline phosphatase staining permits identification of pseudo-lymphoid neuroblasts by their membrane positivity. Finally, the number of cells analysed for each case in this study ($3 \times 10^6$ in two-colour fluorochrome labelling and $6 \times 10^5$ in alkaline phosphatase) allows detection down to $10^{-5}$ neuroblasts by immunological analysis. In this context, two-colour fluorochrome labelling offers several advantages when compared to alkaline phosphatase staining. In the first method, the processing of $3 \times 10^6$ mononuclear cells only requires 90 min once the marrow has been harvested and their analysis on three different slides takes 30 min. Immunostaining and analysis of six smears by the alkaline phosphatase method usually takes 4–5 h and consequently limits the total number of mononuclear cells to be analysed. The greater sensitivity of two-colour fluorochrome labelling is due to the larger number of cells analysed. Two-colour fluorochrome labelling thus appears, both for its sensitivity and its simplicity, as an optimal method to develop routinely in a laboratory involved in clinical programmes in ABMT. The method can be easily adjusted to the analysis of the marrow in various solid tumours. In small cell lung cancer, the three markers used in this study (UJ13A, H11 and 11.14) strongly react with the malignant cells and the method of detection is strictly similar; in other tumours such as breast cancer, relevant markers of the malignant cells are available.

For clinicians, such an analysis clearly selects a group of patients whose autograft is contaminated with malignant cells and for whom the use of a purging procedure is largely justified. In addition, the immunological analysis enables the efficiency of clinical purging procedures to be checked and allows the elimination of malignant cells to be quantified. Such data are of particular interest in a disease such as neuroblastoma in which two patients out of three never reach complete remission and are thus autografted in partial remission. To determine whether relapses are due to the failure of high dose chemotherapy or to the reinjection of malignant cells is a prerequisite in the improvement or modification of therapeutic protocols.

The authors are grateful to Dr G. Janossy (London), Dr J.C. Laurent (Sanofi, Montpellier) and Dr J. Kemshead (London) for providing the monoclonal antibodies. This work was supported by the Association pour la Recherche contre le Cancer (A.R.C., no. 6519), by the Institut National de la Santé et de la Recherche Médicale (INSERM, Réseau LMCE no. 48.60.22) and by the Ligue Départementale de Lutte contre le Cancer, Comité de la Savoie (1987).

### References

ALLAN, P.M., GARSON, J.A., HARPER, E.I. *et al.* (1983). Biological characterization and clinical applications of a monoclonal antibody recognizing an antigen restricted to neuroectodermal tissues. *Int. J. Cancer*, **31**, 591.

AUGUST, C.S., SEROTA, F.T., KOCH, P.A. *et al.* (1984). Treatment of advanced neuroblastoma with supralethal chemotherapy, radiation and allogeneic or autologous marrow reconstitution. *J. Clin. Oncol.*, **2**, 609.

BAYLE, C., ALLARD, T., RODARY, C. *et al.* (1985). Detection of bone marrow involvement by neuroblastoma: comparison of two cytological methods. *Eur. Paediatr. Haematol. Oncol.*, **2**, 123.

BOSTROM, B., NESBIT, M.E. JR & BRUNNING, R.D. (1985). The value of bone marrow trephine biopsy in the diagnosis of metastatic neuroblastoma. *Am. J. Pediatr. Hematol. Oncol.*, **7**, 303.

CHEUNG, N.K.V., VON HOFF, D.D., STRANDJORD, S.E. *et al.* (1986). Detection of neuroblastome cells in bone marrow using $G_{D2}$ specific monoclonal antibodies. *J. Clin. Oncol.* **4**, 363.

COMBARET, V., KREMENS, B., FAVROT, M.C. *et al.* (1988). S-L 11.14: a monoclonal antibody recognizing neuroectodermal tumours with possible value for bone marrow purging before autograft. *Bone Marrow Transplant.* **3**, 221.

D'ANGIO, G.J., AUGUST, C., ELKINS, W. *et al.* (1985). Metastatic neuroblastoma managed by supralethal therapy and bone marrow reconstitution. Results of a four-institution children's cancer study group pilot study, In *Advances in Neuroblastoma Research*, Evans, A., D'Angio, G.J. & Seeger, R.C. (eds) p. 557. Alan R. Liss: New York.

EVANS, A.E., GRIFFIN, G.C., TARTAGLIONE, M. *et al.* (1984). A method of detecting neuroblastoma in human bone marrow by means of two monoclonal antibodies PI 153/J and KE 2. *Hybridoma*, **4**, 289.

FAVROT, M.C., COMBARET, V., COZE, C., PHILIP, I. & PHILIP, T. (1988). Is bone marrow purging efficient and necessary for ABMT in solid tumors? In *Bone Marrow Transplantation: Current Controversies*, Alan R. Liss, Inc., New York (eds): in press.

FAVROT, M.C., FRAPPAZ, D., MARITAZ, O. *et al.* (1986). Histological, cytological and immunological analyses are complementary for the detection of neuroblastoma cells in bone marrow. *Br. J. Cancer*, **54**, 637.

FAVROT, M.C. & HERVÉ, P. (1987). Detection of minimal malignant cell infiltration in the bone marrow of patients with solid tumours, non-Hodgkin lymphomas and leukaemias. *Bone Marrow Transplant.*, **2**, 117.

FAVROT, M., PHILIP, I., COMBARET, V. *et al.* (1987). Experimental evaluation of an immunomagnetic bone marrow purging procedure using the Burkitt lymphoma model. *Bone Marrow Transplant.* **2**, 59.

FRANKLIN, I.M. & PRITCHARD, J. (1983). Detection of bone-marrow invasion by neuroblastoma is improved by sampling two sites with both aspirates and trephine biopsies. *J. Clin. Pathol.*, **36**, 1215.

GRAHAM-POLE, J., LAZARUS, H.M., HERZIG, R.H. *et al.* (1984). High dose melphalan therapy for the treatment of children with refractory neuroblastoma and Ewing sarcoma. *Am. J. Pediatr. Hematol. Oncol.*, **6**, 17.

HARTMANN, O., BENHAMOU, E., BEAUJEAN, F. *et al.* (1987). Repeated high dose chemotherapy followed by purged autologous bone marrow transplantation as consolidation therapy in metastatic neuroblastoma. *J. Clin. Oncol.*, **5**, 1205.

HERCEND, T., REINHERZ, E.L., MEUER, S.C. *et al.* (1983). Phenotypic and functional heterogeneity of human cloned natural killer cell lines. *Nature*, **301**, 158.

HUNTER, R.F., BROADWAY, P., SUN, S. *et al.* (1987). Detection of small cell lung cancer bone marrow involvement by discontinuous gradient sedimentation. *Cancer Res.*, **47**, 2737.

KEMSHEAD, J.T., GOLDMAN, A., FRITSCHY, J. *et al.* (1983). Use of panels of monoclonal antibodies in the differential diagnosis of neuroblastoma and lymphoblastic disorders. *Lancet*, **ii**, 12.

MARITAZ, O., COMBARET, V. & FAVROT, M.C. (1988). Intérêt de l'analyse immunologique pour la détection de neuroblastes résiduels dans la moelle osseuse. *Pathol. Biol.*, **36**, 21.

PHILIP, T., BERNARD, J.L., ZUCKER, J.M. *et al.* (1987*a*). High-dose chemoradiotherapy with bone marrow transplantation as consolidation treatment in neuroblastoma: an unselected group of stage IV patients over 1 year of age. *J. Clin. Oncol.*, **5**, 266.

PHILIP, T., CHAUVIN, F., MICHON, J. *et al.* (1988). A pilot study of double ABMT in advanced neuroblastoma (32 patients). In *4th Int. Conference on Autologous Bone Marrow Transplantation*, Houston K. Dicke, G. Spitzer and S. Jagañath (eds), in press.

PHILIP, T., GHALIE, R., PINKERTON, R. *et al.* (1987*b*). A phase II study of high dose cisplatin and VP16 in neuroblastoma: a report from the Société Française d'Oncologie Pédiatrique. *J. Clin. Oncol.*, **5**, 941.

PRITCHARD, J., McELWAIN, T.J. & GRAHAM-POLE, J. (1982). High dose melphalan with autologous bone marrow rescue for treatment of advanced neuroblastoma. *Br. J. Cancer*, **48**, 86.

TRELEAVEN, J.G., GIBSON, F.M., UGELSTAD, J. *et al.* (1984). Removal of neuroblastoma cells from bone marrow with monoclonal antibodies conjugated to magnetic microspheres. *Lancet*, **i**, 70.

WARNKE, R.A., GATTER, K.C., FALINI, B. *et al.* (1983). Diagnosis of human lymphoma with monoclonal antibodies. *N. Engl. J. Med.*, **309**, 1275.